# C/EBPβ as a master regulator of inflammasome signaling in neurodegenerative diseases: mechanisms and therapeutic implications

**DOI:** 10.3389/fimmu.2025.1656165

**Published:** 2025-09-05

**Authors:** Ji Wang, Yuan Li, Yiyuan Xia

**Affiliations:** ^1^ Hubei Key Laboratory of Cognitive and Affective Disorders, Jianghan University, Wuhan, Hubei, China; ^2^ School of Fine Arts and Design, Hunan City University, Yiyang, Hunan, China; ^3^ Postdoctoral Mobile Station of Journalism and Communication, Hunan Normal University, Changsha, Hunan, China; ^4^ Hubei Provincial Demonstration Center for Experimental Medicine Education, School of Medicine, Jianghan University, Wuhan, Hubei, China

**Keywords:** neuroinflammation, inflammasome activation, C/EBPβ isoforms, neurodegenerative diseases, therapeutic targeting

## Abstract

CCAAT/enhancer-binding protein beta (C/EBPβ), a key transcription factor, plays a central role in regulating inflammasome signaling in neurodegenerative diseases (NDs). This review synthesizes the mechanisms by which C/EBPβ modulates neuroinflammation and its potential as a therapeutic target. We conducted a comprehensive systematic review spanning January 1995 to June 2025, systematically querying Google Scholar and PubMed with the following keywords: neuroinflammation, inflammasome activation, C/EBPβ, therapeutic targeting, and neurodegenerative diseases. C/EBPβ exists in three isoforms-LAP1, LAP2, and LIP-each with distinct functions in inflammasome activation. In Alzheimer’s disease (AD), C/EBPβ drives tau cleavage and Aβ pathology through the AEP axis and exacerbates neuroinflammation by upregulating APOE4. In Parkinson’s disease (PD), C/EBPβ silencing reduces α-synuclein aggregation and dopaminergic neuron loss by suppressing the NLRP3 inflammasome. In Amyotrophic Lateral Sclerosis (ALS), C/EBPβ is hypothesized to contribute to TDP-43-associated inflammasome activation, though this requires further validation. In Multiple Sclerosis (MS), C/EBPβ may influence microglial activation and neuroinflammation, as shown in experimental autoimmune encephalomyelitis models. Modulators of the C/EBPβ-inflammasome axis include endogenous regulators like gut-derived metabolites and pharmacological interventions such as small-molecule inhibitors. Therapeutic strategies targeting C/EBPβ hold promise for mitigating neuroinflammation and neurodegeneration, though challenges remain in achieving isoform-specific targeting and blood-brain barrier penetration. Future directions include CRISPR-based editing and biomarker development for personalized therapies.

## 1 ​​Introduction​​

### 1.1 Overview of neuroinflammation and inflammasomes in neurodegenerative diseases

Neurodegenerative diseases (NDs), including Alzheimer’s disease (AD), Parkinson’s disease (PD), amyotrophic lateral sclerosis (ALS), and multiple sclerosis (MS), are characterized by progressive neuronal loss and functional decline in the central nervous system (CNS). A hallmark shared across these disorders is chronic neuroinflammation, driven by dysregulated immune responses and sustained activation of innate immune pathways (1–3). Central to this process are ​​inflammasomes​​, multiprotein complexes that orchestrate inflammatory signaling and contribute to neuronal damage (4).

Neuroinflammation initially serves as a protective mechanism aimed at eliminating pathogens and cellular debris. Resident CNS immune cells, such as microglia and astrocytes, detect danger-associated molecular patterns (DAMPs) or pathogen-associated molecular patterns (PAMPs) through pattern recognition receptors (PRRs), including Toll-like receptors (TLRs) and Nod-like receptors (NLRs) (5). Acute activation of these pathways promotes tissue repair and homeostasis. However, persistent stimuli-such as misfolded protein aggregates (e.g., amyloid-β [Aβ] in AD, α-synuclein in PD), oxidative stress, or mitochondrial dysfunction-trigger chronic neuroinflammation. This leads to the sustained release of pro-inflammatory cytokines (e.g., IL-1β, IL-18), chemokines, and reactive oxygen species (ROS) (6–8). This chronic state exacerbates neuronal death, synaptic dysfunction, and blood-brain barrier (BBB) disruption, creating a self-perpetuating cycle of neurodegeneration (9–13).

In AD, the presence of abnormally phosphorylated tau protein and extracellular deposits of Aβ peptide are key pathological features (14). These deposits activate microglia and astrocytes, leading to the release of pro-inflammatory cytokines and neurotoxicity (15–17). In PD, the misfolding and aggregation of α-synuclein due to oxidative stress result in the accumulation of toxic protein aggregates (18, 19). This triggers a cascade of pro-inflammatory events in microglia and astrocytes, amplifying neuronal loss and persistent neurodegeneration (20, 21).

The NLRP3 inflammasome is a critical player in neuroinflammation, activated by DAMPs and PAMPs in microglia, astrocytes, and neurons (22–24). Activation of the NLRP3 inflammasome leads to the maturation and release of pro-inflammatory cytokines such as IL-1β and IL-18, contributing to neuroinflammation and neuronal damage (25). Inhibiting the NLRP3 inflammasome has been proposed as a potential therapeutic strategy to counteract neurodegenerative diseases (1, 26). Most evidence and therapeutic concepts presented here derive from *in vitro* systems or animal models. While indispensable for mechanistic insight and proof-of-concept, these data must be validated in rigorously designed clinical trials before any therapeutic claims can be extended to human disease.

### 1.2 Introduction to C/EBPβ: structure, isoforms, and physiological roles

CCAAT/enhancer-binding protein beta (C/EBPβ) is a member of the C/EBP family of transcription factors that play crucial roles in various physiological processes (27). As a leucine-zipper (bZIP) transcription factor, C/EBPβ binds DNA as dimers and regulates the transcription of genes containing specific T(TG)NNGNAA(TG) motifs (28, 29). The uniqueness of the C/EBPβ gene lies in its lack of introns and its ability to be alternatively translated into three major isoforms: liver activator protein 1(LAP1), liver activator protein 2(LAP2), and liver inhibitor protein (LIP) (30). These isoforms arise through alternative translation initiation sites, leading to differences in their N-terminal regions (30, 31).

LAP1 and LAP2 are both transcriptional activators, with LAP2 being a stronger transactivator than LAP1. This difference is attributed to the regulation of C/EBPβ protein tertiary structure and unique N-terminal protein-protein interactions (28, 32). LAP1 and LAP2 differ in their first 21 N-terminal amino acids due to internal translation initiation from the downstream LAP2 start codon (28, 30). In contrast, the LIP isoform lacks the N-terminal transactivation domain (TAD) and most of the negative regulatory domain, functioning primarily as a dominant-negative regulator of transcription. However, in some cellular contexts, LIP can act as a transcriptional activator by interacting with other cofactors (30, 33).

C/EBPβ is expressed in various tissues, including the liver, brain, intestine, and skin, and is involved in multiple physiological processes (34). It is essential for the differentiation of mammary epithelial and granulosa cells, macrophage function, and brown adipose tissue formation (35–37). C/EBPβ also plays a role in cell cycle arrest and differentiation in hepatocytes, hematopoietic cells, and adipocytes (37–39). Additionally, it is involved in apoptosis and senescence in microglia and neurons. For instance, methamphetamine (METH) upregulates C/EBPβ expression, thereby activating Lipocalin2 (an apoptosis-inducing factor) and leading to apoptosis in microglial cells. Silencing C/EBPβ can reverse this process (40, 41). METH induces neuronal autophagy and apoptosis through the C/EBPβ-DDIT4/TSC2/mTOR signaling axis and the Trib3/Parkin/α-syn pathway. Inhibition of C/EBPβ can mitigate neurotoxicity (42, 43). C/EBPβ regulates the pro-inflammatory program in microglia and is involved in the expression of several inflammatory genes in astrocytes (44, 45). It also plays a role in the regulation of the complement component 3 gene in neural cells, contributing to its pro-inflammatory effects (46).

### 1.3 Rationale for focusing on C/EBPβ-inflammasome axis: bridging transcriptional regulation and chronic inflammation in NDs

The C/EBPβ-inflammasome axis has emerged as a critical pathway linking transcriptional regulation to chronic inflammation in NDs (**Table 1**). This axis is particularly relevant in conditions such as AD, PD, ALS, and MS, where chronic neuroinflammation plays a significant role in disease progression (54).

**Table 1:** The molecular pathways of neurodegenerative diseases are regulated by C/EBPβ.

Diseases	Mechanisms	Reference
AD	Glia activation leads to increased C/EBPβ expression, its nuclear translocation, and binding to pro-inflammatory gene promoters, thereby upregulating inflammatory genes.	(46)
	C/EBPβ binds the promoter of APOE and escalates its expression	(47)
	Neuronal ApoE4 activates C/EBPβ and promotes δ-secretase simultaneously cleaves both APP and Tau and augments Aβ production and Tau hyperphosphorylation.	(48, 49)
	Diabetes-linked inflammation triggers neuronal C/EBPβ activation, leading to increased APP and Tau expression.	(50)
PD	C/EBPβ acts as a transcription factor to upregulate α-syn and monoamine oxidase B, which triggers oxidative stress in dopaminergic neurons and α-Syn aggregation.	(51)
	Disruption of gut microbiota homeostasis and impairment of the intestinal barrier activate the C/EBP/AEP signaling, leading to α-synuclein aggregation and substantia nigra dopaminergic neuron degeneration.	(52)
MS	Myelin basic protein (MBP)-specific T cells trigger microglial inflammation via a C/EBPβ-dependent mechanism.	(53)

C/EBPβ plays a pivotal role in regulating the expression of genes involved in inflammasome activation, thereby linking transcriptional regulation to chronic inflammation. Inflammasomes, such as the NLRP3 inflammasome, are activated by various stimuli, including misfolded protein aggregates, oxidative stress, and mitochondrial dysfunction (55–58). For instance, C/EBPβ has been shown to directly bind to the promoter region of the SerpinB2 gene, which is crucial for LPS-induced transcription in macrophages. This binding is essential for driving transcription from the SerpinB2 promoter in response to LPS stimulation (59). Additionally, C/EBPβ regulates the expression of caspase-1, a key component of the non-canonical inflammasome pathway (60, 61). This regulation is critical for understanding how chronic inflammation is sustained in NDs.

C/EBPβ is also implicated in the regulation of mitochondrial function and the expression of mitochondrial transcription factor A (TFAM). In a cellular model of Parkinson’s disease using SH-SY5Y dopaminergic cells treated with the neurotoxin 6-hydroxydopamine (6-OHDA), C/EBPβ levels increased over time, reaching a peak at 18 hours when cells began to die due to stress. In contrast, TFAM expression decreased after 4 hours of treatment, followed by a partial recovery. This recovery is likely due to C/EBPβ’s activation of the TFAM promoter (62). Mitochondrial dysfunction, a common feature of NDs, contributes to chronic inflammation through the release of damage-associated molecular patterns (DAMPs) (63, 64). By regulating mitochondrial function, C/EBPβ can influence the inflammatory response and contribute to the pathogenesis of NDs.

C/EBPβ has been shown to regulate autophagy, a process crucial for the degradation of damaged mitochondria. In C/EBPβ-silenced cells, there is an accumulation of autophagic markers under oxidative stress and inflammatory conditions, indicating that C/EBPβ is involved in the regulation of autophagy. This accumulation is not due to increased autophagy induction but rather to decreased autophagosome degradation (62, 65). This finding suggests that C/EBPβ may play a role in maintaining the balance between mitochondrial biogenesis and degradation, which is essential for cellular homeostasis.

## 2 ​​C/EBPβ in inflammasome activation​​

### 2.1 Transcriptional control of inflammasome components​​

**Figure 1:**
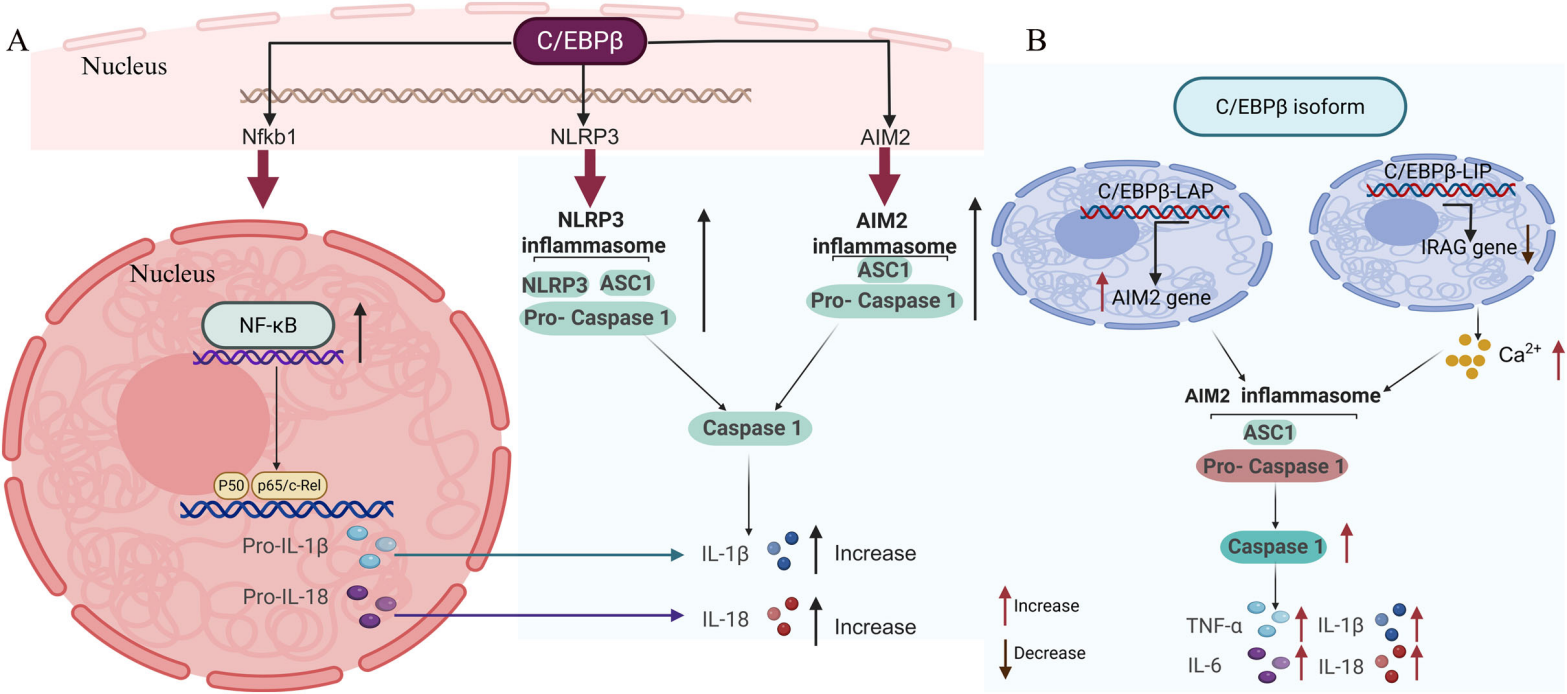
Transcriptional regulation of inflammasome activation by C/EBPb. C/EBPb governs the expression of NLRP3 and AIM2, which are pivotal inflammasome components responsible for triggering the cleavage of latent procaspase-1 into its active form, caspase-1. This activation facilitates the conversion of the cytokine precursors pro-IL-1b and pro-IL-18 into their mature, bioactive forms, IL-1b and IL-18, respectively. Beyond this, C/EBPb directly regulates caspase-1 expression and influences NF-kB expression, which in turn modulates the activation of pro-IL-1b and pro-IL-18. (B) The C/EBPb isoforms, C/EBPb-LAP and C/EBPb-LIP, drive the production of inflammatory factors by activating the AIM2 inflammasome. Created with BioRender.com.

C/EBPβ is a key transcription factor that directly regulates the expression of critical inflammasome components by binding to their promoter regions. This direct regulation is essential for the expression and activation of inflammasomes, which play a crucial role in the inflammatory response in various diseases (66). For instance, C/EBPβ has been shown to regulate the expression of NLRP3, a key component of the inflammasome complex (60, 67). NLRP3 is activated by various stimuli, including misfolded proteins and oxidative stress, leading to the release of pro-inflammatory cytokines like IL-1β and IL-18 (68–70). Similarly, C/EBPβ also regulates the expression of AIM2, another inflammasome sensor that recognizes cytosolic DNA and forms a caspase-1-activating inflammasome (71, 72). Additionally, C/EBPβ directly controls the expression of caspase-1, a crucial enzyme in the inflammasome pathway that processes pro-IL-1β and pro-IL-18 into their active forms (60). The specific mechanism is illustrated in **Figure 1A**.

C/EBPβ not only acts independently but also synergizes with other transcription factors, such as NF-κB, to amplify the production of pro-inflammatory cytokines (**Figure 1A**). This synergy is particularly evident in the regulation of IL-1β and IL-18, two key cytokines involved in the inflammatory response (73, 74). NF-κB is a well-known regulator of inflammatory genes, and its cooperation with C/EBPβ enhances the transcriptional activation of these genes. For example, studies have shown that C/EBPβ and NF-κB can bind to adjacent sites on the promoters of IL-1β and IL-18 genes, leading to a coordinated upregulation of these cytokines (60). This synergistic effect is crucial for the robust inflammatory response observed in conditions such as sepsis and neurodegenerative diseases (46, 62, 75, 76).

### 2.2 Isoform-specific mechanisms​

C/EBPβ-LAP, the transcriptionally active isoform of C/EBPβ, is crucial for activating the AIM2 inflammasome by enhancing the expression of AIM2 and caspase-1 (**Table 2**). This mechanism has been observed in several contexts.

**Table 2:** The role of the C/EBPβ isoform in inflammation.

C/EBPβ isoforms					Reference
Structural Features		LAP1	LAP2	LIP	
Full-length N-terminal transactivation domain (TAD)	Lacking 21 amino acids at the N-terminal TAD and partially regulatory	Completely lacking the N-terminal TAD and Lacking most of the negative regulatory domain	(30)
Primary Functions		Increase transcriptional activity	Increase transcriptional activity	Inhibit transcriptional activity	(31)
Diseases Mechanisms	Lupus nephritis	C/EBPβ-LAP upregulates AIM2 inflammasome activity by promoting the transcriptional expression of AIM2 and CASPASE1 through direct promoter binding		C/EBPβ-LIP suppresses IRAG expression at the transcriptional level, enhancing Ca²^+^ release and subsequent AIM2 inflammasome activation	(71)
	Chronic inflammation	LAP isoforms promote inflammation by activating the transcription of pro-inflammatory genes, including IL-6 and TNFα		LIP reduces inflammation by blocking LAP	(77)

In Lupus Nephritis (LN), C/EBPβ-LAP activates the AIM2 inflammasome and induces podocyte pyroptosis. This activation is achieved by binding to the promoters of AIM2 and CASPASE1, thereby enhancing their expression (**Figure 1B**). Knockdown of AIM2 or caspase-1 reversed the effects of C/EBPβ-LAP overexpression, highlighting the critical role of these interactions in inflammasome activation (71). In the context of liver inflammation, C/EBPβ-LAP activates the transcription of various pro-inflammatory genes, including IL-6 and TNF. This activation is crucial for the acute phase response and the recruitment of immune cells to the site of inflammation (66, 77, 78).

In contrast to C/EBPβ-LAP, the C/EBPβ-LIP isoform functions primarily as a transcriptional inhibitor and has been shown to promote Ca²^+^-mediated inflammasome assembly by suppressing the expression of inositol-1,3,4-phosphat receptor associated G-kinase substrate (IRAG). Overexpression of C/EBPβ-LIP in LN transcriptionally inhibits IRAG, leading to increased Ca²^+^ release. This increase in Ca²^+^ levels facilitate the assembly and activation of the AIM2 inflammasome (71, 79, 80). This finding suggests that C/EBPβ-LIP not only regulates the expression of key inflammasome proteins but also affects their polymerization through the regulation of Ca²^+^ release (**Figure 1B**). In Myeloid-Derived Suppressor Cells (MDSCs), C/EBPβ-LIP suppresses the expression of immunosuppressive genes such as arginase-1 (Arg-1) and inducible nitric oxide synthase (iNOS). This suppression is achieved by blocking the activity of the transcriptionally active LAP-1 and LAP-2 isoforms (78). The balance between LIP and LAP isoforms is crucial for the regulation of MDSC function and the inflammatory response. In hepatocytes, C/EBPβ-LIP has been shown to downregulate the expression of cytochrome P450 enzymes (e.g., CYP3A4) by antagonizing the transactivation activity of LAP. This mechanism involves the binding of LIP to LAP (81, 82), preventing it from initiating transcription. This regulation is important for the metabolic response to inflammatory stimuli.

## 3 ​​Role in specific neurodegenerative diseases​​

### 3.1 Alzheimer’s disease

C/EBPβ has been identified as a key player in the progression of AD. Over the past decade, numerous studies have elucidated its multifaceted role in the pathogenesis of AD. For instance, C/EBPβ has been shown to be a crucial transcription factor for APOE, preferentially mediating the expression of ApoE4, which is associated with an increased risk of AD (47). In addition to its role in Aβ production, C/EBPβ is implicated in the neurofibrillary pathology of AD. It has been demonstrated that C/EBPβ can upregulate the expression of certain proteins that mediate the cleavage of tau and APP, proteins implicated in the development of AD (48, 49). This suggests that C/EBPβ may contribute to both major pathological features of AD.

Moreover, C/EBPβ is involved in neuroinflammation, a critical component of AD pathology (83, 84). It is a key regulator of pro-inflammatory genes in microglia and is overexpressed in AD animal models and AD patients. There is a positive feedback loop between C/EBPβ and inflammatory components-inflammatory factors can activate C/EBPβ, which in turn further promotes the production of inflammatory factors (46, 66). This inflammatory response can further exacerbate neuronal damage and contribute to the progression of the disease. Chronic neuroinflammation activates the transcription factor C/EBPβ, which in turn up-regulates the cysteine protease asparagine endopeptidase (AEP) (85). Clinically, heightened AEP activity is documented in post-mortem AD brains, while mechanistic studies reveal that genetic ablation of C/EBPβ attenuates AD pathology via AEP suppression in animal models (86, 87). Importantly, AEP truncates tau at N368 and N255, yielding aggregation-prone fragments that precipitate neurofibrillary tangle formation—a defining feature of AD neurodegeneration (88).

In AD mouse models, knockdown of C/EBPβ significantly reduces the levels of inflammatory factors and the number of activated microglia. Conversely, overexpression of C/EBPβ exacerbates these pathological features (48). C/EBPβ isoforms can bind the promoter regions of inflammasome genes (e.g., caspase-1, NLRP3, and AIM2) via their DNA-binding domains and enhance transcription. While this suggests a potential regulatory role in inflammasome-mediated processes in AD, direct experimental confirmation is still lacking.

Recent studies have also highlighted the potential therapeutic implications of targeting C/EBPβ. For example, inhibiting the C/EBPβ/δ-secretase axis has been shown to reduce Aβ levels and improve cognitive function in animal models of AD (89). Decrease FOXO inhibition, reverse GABA neuron degeneration, maintaining the homeostasis of excitation inhibition balance (90). These findings suggest that interventions aimed at modulating C/EBPβ activity could represent a promising strategy for treating AD (**Table 1**).

### 3.2 ​​Parkinson’s disease

Over the past decade, research has illuminated the multifaceted role of C/EBPβ in PD (51, 62). C/EBPβ is involved in the regulation of AEP, also known as legumain, a cysteine protease highly activated in the brains of PD patients (91). AEP cleaves α-synuclein (α-syn), promoting its aggregation and neurotoxicity, which contributes to the loss of dopaminergic neurons and motor deficits characteristic of PD (52, 92). Additionally, C/EBPβ acts as a transcription factor to upregulate α-syn and monoamine oxidase B (MAOB), both of which are implicated in PD pathogenesis in human wild-type α-Syn transgenic mice (51). This transcription factor can be activated by lipopolysaccharide (LPS) and inflammatory cytokines such as interleukin-1β (IL-1β), IL-6, and tumor necrosis factor-α (TNF-α) (50). Therefore, gut microbiota dysbiosis and inflammation activation contribute to PD pathology through the C/EBPβ/AEP signaling pathway (52). In a study using a rotenone-induced PD mouse model, combined with antibiotic-induced microbiome depletion and fecal microbiota transplantation, it was found that gut microbiota dysbiosis, along with leaky gut-induced bacterial endotoxins, activates C/EBPβ/AEP signaling and α-syn pathology, ultimately leading to neurodegeneration in PD (52). This suggests that the gut microbiota may play a significant role in the activation of C/EBPβ/AEP signaling and the progression of PD.

Furthermore, silencing C/EBPβ has been shown to reduce α-synuclein aggregation and dopaminergic neuron loss. This effect is mediated through the suppression of the NLRP3 inflammasome (93, 94). By downregulating C/EBPβ, the expression of NLRP3 and other inflammasome components is reduced, thereby attenuating the inflammatory response and mitigating neuronal damage in an MPTP neurotoxic model of PD (55, 95). This finding suggests that targeting C/EBPβ could be a promising therapeutic strategy for reducing neuroinflammation and neurodegeneration in PD (**Table 1**).

### 3.3 ​​Amyotrophic lateral sclerosis

TDP-43 (TAR DNA-binding protein 43) is a nuclear protein that regulates several RNA metabolic pathways. Dysregulation of TDP-43 induces its cytoplasmic accumulation and aggregation, which is a hallmark of ALS (96). The C/EBPβ expression in microglia has indeed been observed to increase in spinal cord of ALS animal models and human ALS patients (97).TDP-43 interacts with NF-κB, a key factor contributing to the inflammatory response, and activates NF-κB in microglia (98). Activated NF-κB induces the production of pro-inflammatory cytokines, contributing to neuroinflammation (97, 99). Considering that C/EBPβ is a key transcription factor for NF-κB,it may play a role in regulating these inflammatory pathways, thereby contributing to the neuroinflammatory response in ALS (100).

### 3.4 Multiple sclerosis

C/EBPβ has been shown to be involved in the inflammatory response in MS. Specifically, myelin basic protein-specific T cells, an autoantigen in MS, induce the expression of IL-1β, IL-1α, TNF-α, and IL-6 in microglial cells through a mechanism dependent on C/EBPβ activation (53). This suggests that C/EBPβ plays a crucial role in mediating the inflammatory response in MS (53). In the context of MS, C/EBPβ may contribute to the activation of the NLRP3 inflammasome, which is associated with the production of IL-1β and IL-18, key cytokines in neuroinflammation (101). This activation can lead to further recruitment of immune cells and exacerbation of the inflammatory process in MS. Additionally, studies have shown that C/EBPβ deficiency in myeloid cells can reshape microglial gene expression and is protective in experimental autoimmune encephalomyelitis (EAE), a mouse model of MS (102). However, additional direct evidence is necessary to demonstrate that C/EBPβ regulates the inflammasome in MS pathogenesis. Furthermore, C/EBPβ has been implicated in the regulation of other inflammatory mediators such as HMGB1. HMGB1, a damage-associated molecular pattern (DAMP) protein, is known to interact with various receptors including RAGE, TLR2, TLR4, and TLR9, and can induce inflammatory responses (103–105), C/EBPβ can be activated by inflammatory stimuli such as lipopolysaccharide (LPS), and it has been shown to upregulate the expression of IL-1β, a cytokine that can be further enhanced by HMGB1 through its interaction with transcription factors like PU.1 (106–109). This suggests that C/EBPβ and HMGB1 can act in concert to amplify inflammatory signaling.

## 4 ​​Modulators of the C/EBPβ-inflammasome axis​​

### 4.1 Endogenous regulators​​

Gut-derived metabolites, such as 12-HHTrE, have been identified as activators of the C/EBPβ-inflammasome axis. These metabolites can induce oxidative stress, which in turn activates C/EBPβ and promotes the expression of pro-inflammatory cytokines (110). Additionally, oxidative stress itself can act as an activator of this axis, contributing to the production of reactive oxygen species (ROS) and subsequent inflammatory responses (111–113). On the other hand, COP1-mediated ubiquitination and degradation of C/EBPβ in microglia have been reported as a mechanism to control the activity of this transcription factor (114). This process helps to regulate the inflammatory response by reducing the levels of active C/EBPβ, thereby limiting the activation of the inflammasome.

### 4.2 ​​Pharmacological interventions​​

Small-molecule inhibitors have been developed to target components of the C/EBPβ-inflammasome axis (115, 116). For instance, an AEP inhibitor, Compound #11A, has been shown to reduce the activation of the inflammasome and mitigate neuroinflammation (117, 118). This pharmacological approach aims to block the downstream effects of C/EBPβ activation, thereby reducing the production of pro-inflammatory cytokines. Among pharmacologic NLRP3 inhibitors, the orally bioavailable agent ZYIL1 prevents ASC oligomerization; its Phase II trial in ALS has recently concluded. Likewise, VTX3232—an orally active, CNS-penetrant, and selective NLRP3 inhibitor—is currently undergoing Phase II evaluation in early-stage Parkinson’s disease (119).

Another strategy involves the use of lentiviral shRNA delivery systems to silence C/EBPβ expression. This method has been employed to specifically target and reduce the levels of C/EBPβ in cells, thereby attenuating the activation of the inflammasome and associated inflammatory responses (120, 121). This approach provides a potential therapeutic avenue for conditions where excessive activation of the C/EBPβ-inflammasome axis contributes to pathology.

## 5 ​​Therapeutic implications and challenges​​

### 5.1 Preclinical success​​

Knockdown of C/EBPβ has shown promising results in preclinical models of AD and PD. Studies have demonstrated that reducing C/EBPβ levels can improve cognitive function and reduce pathological markers in these models. For instance, in PD models, C/EBPβ reduction has been shown to mitigate dopaminergic neuronal damage and glial activation, suggesting that C/EBPβ depletion could be a valuable therapeutic target for PD (52). Similarly, in AD models, the anti-inflammatory cytokine interferon-gamma acts as a potential therapeutic target of AD (122), and C/EBPβ knockdown has been associated with reduced amyloid-beta (Aβ) pathology and improved cognitive outcomes (47).

### 5.2 Translational barriers

Despite these encouraging preclinical findings, several translational barriers must be addressed to develop effective therapies targeting the C/EBPβ-inflammasome axis. One significant challenge is achieving isoform-specific targeting of C/EBPβ to avoid off-target effects. C/EBPβ has multiple isoforms, and targeting the wrong isoform could lead to unintended consequences (47, 123). Therefore, developing strategies to specifically target the pathogenic isoforms of C/EBPβ is crucial. High-throughput small-molecule screening can now be coupled with AI/ML-driven chemical-structure modelling, similarity-based target prediction, and cross-species transcriptomics to markedly reduce off-target liabilities.

Another major challenge is the blood-brain barrier (BBB) penetration for inhibitors. The BBB restricts the entry of many drugs into the central nervous system, making it difficult to deliver effective concentrations of therapeutic agents to the brain (124). Overcoming this barrier is essential for the successful translation of C/EBPβ inhibitors from preclinical to clinical settings. Nanobodies—single-domain antibodies of ~15 kDa—readily access cryptic epitopes inaccessible to conventional antibodies and can stabilize distinct protein conformations with exquisite specificity. Several nanobodies have already demonstrated brain penetrance (125) and the platform is rapidly gaining traction as a therapeutic modality for CNS disorders (126, 127).

### 5.3 Future directions

Looking forward, several innovative approaches hold promise for addressing these challenges. CRISPR-based isoform editing is a cutting-edge technology that could enable precise targeting of specific C/EBPβ isoforms, thereby reducing the risk of off-target effects (128–130). This approach could be particularly useful in selectively targeting the pathogenic isoforms of C/EBPβ in neurodegenerative diseases.

Additionally, the development of biomarkers for C/EBPβ activity could facilitate patient stratification and personalized medicine. Identifying reliable biomarkers that reflect C/EBPβ activity *in vivo* would allow for better selection of patients who are most likely to benefit from C/EBPβ-targeted therapies (131). This could enhance the success rate of clinical trials and improve patient outcomes.

## 6 Conclusion

In conclusion, the C/EBPβ-inflammasome axis provides a critical link between transcriptional regulation and chronic inflammation in NDs. Understanding this axis is essential for developing therapeutic strategies aimed at mitigating neuroinflammation and neurodegeneration. Future research should focus on elucidating the specific mechanisms by which C/EBPβ regulates inflammasome activation and identifying potential therapeutic targets within this axis.

C/EBPβ, a transcription factor, plays a vital role in regulating immune and inflammatory responses. It has been shown to directly target the promoter region of various genes, such as IL-1β, thereby contributing to the activation of the NLRP3 inflammasome (106, 132). This activation leads to the production of pro-inflammatory cytokines, which are key drivers of neuroinflammation in NDs. For instance, in models of Alzheimer’s disease and Parkinson’s disease, C/EBPβ knockdown has been shown to reduce pathological markers and improve cognitive function (133–136).

However, targeting C/EBPβ for therapeutic purposes presents several challenges. One major issue is achieving isoform-specific targeting to avoid off-target effects, as C/EBPβ has multiple isoforms with distinct functions. Additionally, delivering therapeutic agents across the blood-brain barrier remains a significant hurdle, as the BBB restricts the entry of many drugs into the central nervous system.

Future directions in research should include the development of isoform-specific inhibitors and strategies to enhance BBB penetration. CRISPR-based isoform editing could enable precise targeting of specific C/EBPβ isoforms, thereby reducing the risk of off-target effects. Moreover, the development of biomarkers for C/EBPβ activity could facilitate patient stratification and personalized medicine, allowing for better selection of patients who are most likely to benefit from C/EBPβ-targeted therapies.

In summary, the dual role of C/EBPβ as a transcriptional orchestrator and inflammasome amplifier underscores its potential as a therapeutic target for NDs. Emphasis on personalized therapeutic strategies targeting this axis could lead to more effective treatments for mitigating neuroinflammation and neurodegeneration. Future research should aim to overcome current translational barriers and explore innovative approaches to harness the therapeutic potential of targeting the C/EBPβ -inflammasome axis.
